# Cellular tools for biosimilar mAb analysis

**DOI:** 10.1186/1753-6561-7-S6-P31

**Published:** 2013-12-04

**Authors:** Carsten Lindemann, Silke Mayer, Miriam Engel, Petra Schroeder

**Affiliations:** 1 EUFETS GmbH, 55743 Idar-Oberstein, Germany

## Background

For the development of biosimilar monoclonal antibodies (mAb) or related substances containing the IgG Fc part it is mandatory to fully compare immunological properties between originator and biosimilar in a "comparability exercise" [1]. The most complex Fc associated function to mediate antibody dependent cellular cytotoxicity (ADCC) needs to be characterized using the active substance of the biosimilar and the comparator. From a regulatory point of view potency assays should reflect the proposed mode of action but in vitro ADCC assays are considered difficult to validate due to the variability of the primary effector cells [2,3]. The requirement to test for ADCC with high precision and accuracy is challenging. Design of cell lines to replace primary cells for effector or target cells is a solution to provide tools for standardized and extensive biosimilar testing.

## Materials and methods

Retroviral vectors were used to generate cell lines with stable genetic modification. Vector particles were generated by transient transfection of 293T cells with plasmids encoding gag, pol/env and an expression plasmid containing the packaging region and the sequences of promotor and the transgenes, i.e. selection marker and gene of interest. Multiple gene expression was achieved either by using a bicistronic design enabling transcription from two promotor sequences, or by using an internal ribosomal entry site. Transduction of cells in log phase was followed by a selection of transduced cells and clonal selection by limiting dilution. Cell clones were expanded for primary and secondary cell banks and further characterised with regard to transgene expression and functional characteristics. We developed a human transgenic NK-cell line (YTE756.V#26, effector cell line) with stable expression of Fc gamma-receptor IIIA (CD16, high affinity variant, valine at position 159) and stable functional characteristics. Target cell lines were generated similarly using different expression plasmid constructs.

ADCC assays were developed by using design of experiments (DoE) to determine experimental factors of importance for assay suitability. To show assay suitability goodness of fit, the amplitude of sigmoid curve, slope and parallelism was determined for each sample compared to a standard. Hypo- and hyperpotent samples (50%, 100%, 150% and 200% potency) of Rituximab, Trastuzumab, Adalimumab and Infliximab were analysed to determine accuracy and linearity of each method. Optimisation of each assay requires determining the relative importance of factors including E:T ratio, incubation time, target cell density and pre-assay schedules for target and effector cells. Analysis of critical factor interaction was performed using Minitab software. A list of established ADCC assays is shown in Table 1.

**Table 1:** ADCC assay systems

Antibody	Target cell line	Read-out	Selection of cell line
Trastuzumab	HER-2^+ ^SK-OV-3 cells	metabolic activity of residual target cells	selected from various breast cancer cell lines
Rituximab	CD20^+ ^Granta-519 cells	Calcein release by target cells	selected from various hematopoietic tumor cell lines
Cetuximab	EGFR^+ ^SK-OV-3 cells	metabolic activity of residual target cells	selected from various breast cancer cell lines
Infliximab	membraneTNFalpha^+ ^293T cells	Calcein release by target cells	generated by genetic modification
Adalimumab	membraneTNFalpha^+ ^HT1080 cells	metabolic activity of residual target cells	

CD16 expression was analyzed and quantified by flow cytometry. Cells were stained using anti-CD16 PE-conjugated antibodies. PE-fluorescence was correlated to number of PE-molecules per cell using BD Quantibrite beads. Primary NK-cells were isolated using Dynal beads (purity > 95%) from 3 healthy donors and used immediately after isolation.

## Results

In order to prove genetic stability of the transgenic NK cell line CD16 expression was analysed by flow cytometry for up to 22 passages. More than 95% of cells were CD16 positive, viability of cells was >90%. CD16 expression level was stable (19.000 - 28.000 CD16 molecules/cell).

Functional stability of the effector cell line was shown for more than 30 passages. This was shown by a stable EC50 value obtained for a reference antibody in the Trastuzumab ADCC assay.

The effector cell line was compared with primary NK-cells (purity > 95%) from 3 donors in a Trastuzumab ADCC assay. The data show high donor variability, mostly incomplete dose-response curves and a killing activity with a low dynamic range (baseline to top ratio: 3). For primary NK-cells the amplitude of the dose-response curve is dependent on both donor variability and the type of target cell. Using the effector cell line this is dependent on the target cell only. Assay variability was strongly reduced and sample throughput could strongly be increased by using the effector cell line in comparison to primary NK-cells. Optimization of each assay by DoE required determining the relative importance of various factors including effector to target cell ratio, incubation time, target cell density and pre-assay culture schedules for target and effector cells. Accuracy of these ADCC assays could be shown in between a range of 50% to 200% potency. Linearity was shown by a high coefficient of determination (>0.97) and other statistical methods. Inter-assay precision of all ADCC assays was <20%.

ADCC assays for Infliximab and Adalimumab require a membraneTNFalpha expressing target cell line (Table 1). In this fully designed ADCC test system both the transgenic NK-effector cell line and the target cell line were generated by genetic modification. In the presented case, the test system consists of HT1080 target cells modified to express membraneTNFalpha and the transgenic NK-cell line.

Accuracy and linearity of the Infliximab ADCC assay was analysed by measuring items containing varying theoretical antibody concentrations to simulate hypo-potent and hyper-potent samples. Linearity was shown by a high coefficient of determination or by testing if the 2nd order polynomial model is non-significant (0 is included in the 95% confidential interval of B2). For precision analysis the relative potency of a sample was repeatedly analyzed on 4 days with 3 assays per day.

## Conclusions

Altogether these data show the feasibility of providing suitable tools for validation and routine testing of various mAbs in ADCC potency assays scalable to the analytical needs of biosimilar testing.

**Figure 1:**
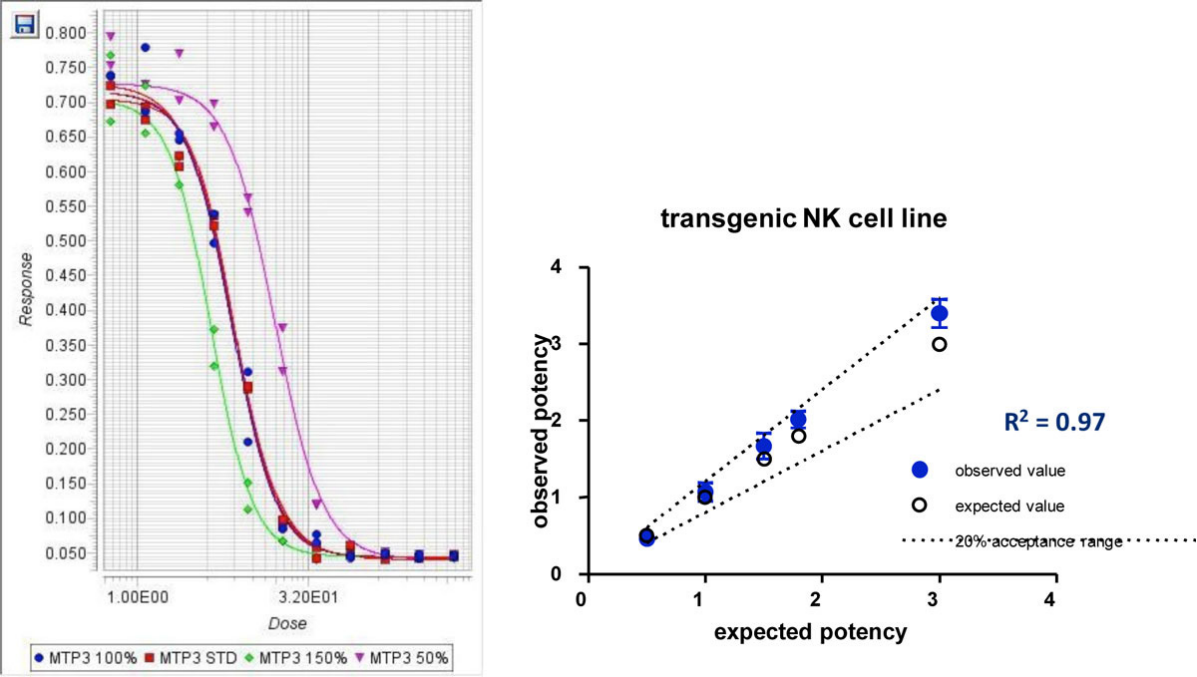
**Analysis of accuracy and linearity of the Infliximab ADCC assay**. Data shown are sample dose response curves (left) determined by 4PL analysis and mean rel. potency +/- SD (dot, n = 3) compared to the standard (right).
